# Supplementary material for: Agentic genomics: From pipeline automation to autonomous validation
Source: Cell Genom. 2026 Jul 21;6(8):101305. doi: 10.1016/j.xgen.2026.101305 (PMC13477014; doi:10.1016/j.xgen.2026.101305)
Supplement: Supplementary file 1 [file mmc1.pdf]

Cell Genomics, Volume *6*

Supplemental information

Agentic genomics: From pipeline automation to autonomous validation

Manuel Corpas, Heinner Guio, and Segun Fatumo

Supplemental Information

Agentic Genomics: From Pipeline Automation to Autonomous Validation

Manuel Corpas, Segun Fatumo, Heinner Guio

This document provides supplemental material referenced in the main text: a key resources table (Table S1), the full description of the perturbation test inputs (Note S1), and notes on the basis of the qualitative syntheses presented in Box 1 and Tables 1 and 3 (Notes S2 and S3).

Table S1. Key resources.

Resource	Source	Identifier
ClawBio skill library	Corpas (2026)	https://doi.org/10.5281/zenodo.19420648
pharmgx-reporter skill (perturbation test)	ClawBio	version 0.1.0

Note S1. Perturbation test inputs.

The perturbation result reported in the validation section of the main text was executed on 2 June 2026 using ClawBio's pharmgx-reporter skill (version 0.1.0). The skill was run on three perturbed inputs and, as a control, one valid input:

1. An empty file (zero bytes, no genomic data).
2. A file containing non-genotype content (text that does not conform to any recognised genotype format).
3. A comment-only file with header or comment lines but no variant rows.
4. As a control, one valid 23andMe-format genotype file.

Each perturbed input caused the skill to halt with an explicit file-format detection error and a non-zero exit status, whereas the valid input produced a complete report. This reproduces, on the current release, the perturbation that an earlier community audit found to produce a silent "all normal" pharmacogenomic report. The silent-failure path exposed by the original audit has been closed by the governance-layer remediation process described in the main text: community audit, public disclosure, and a fix that converts a silent degradation into a loud, diagnosable failure.

Note S2. The Box 1 illustrative example.

Box 1 in the main text is an illustrative example, not a benchmark or a logged experiment. It describes, qualitatively, the pattern we have observed when an AI agent orchestrates standard open-source tools (fastp for adapter trimming and quality control, BWA-MEM2 for alignment to GRCh38, GATK HaplotypeCaller and DeepVariant for variant calling, and SnpEff with ClinVar for annotation) on a local workstation for trio exome analysis. We did not conduct a systematic benchmark for this Perspective; Box 1 reports no timings, counts, or comparative measurements, and no claim of measured performance is made. Controlled, logged, multi-site benchmarking with confidence intervals is identified in the main text as a priority for the field rather than a result of this work.

Note S3. Tables 1 and 3.

The values in Table 1 are a qualitative synthesis drawn from the cited reports and contain no new measurements. The tier classification in Table 3 was derived solely from the validation evidence described in each system's cited publication, with each system placed at the highest tier for which published evidence exists.
