# Supplementary material for: Agentic genomics: From pipeline automation to autonomous validation
Source: Cell Genom. 2026 Jul 21;6(8):101305. doi: 10.1016/j.xgen.2026.101305 (PMC13477014; doi:10.1016/j.xgen.2026.101305)
Supplement: Supplementary file 2 [file mmc2.pdf]

Cell Genomics Transparent Peer Review Record

Agentic Genomics: From Pipeline Automation to Autonomous Validation

Author list: Manuel Corpas, Segun Fatumo, Heinner Guio

Summary

Initial submission:	Received : April 4th 2026
	Scientific editor: Judith Nicholson
First round of review:	Number of reviewers: 2<br>Revision invited : May 26th 2026<br>Revision received : June 3rd 2026
Second round of review:	Number of reviewers: 1<br>Accepted : June 25th 2026
Data freely available:	N/A
Code freely available:	N/A

This transparent peer review record is not systematically proofread, type-set, or edited. Special characters, formatting, and equations may fail to render properly. Standard procedural text within the editor's letters has been deleted for the sake of brevity, but all official correspondence specific to the manuscript has been preserved.

Referees' reports, first round of review

Reviewer 1:

This is a timely and forward looking Perspective discussing the opportunities and challenges of agentic bioinformatics workflows. It (correctly) argues that agentic workflows will shift the emphasis of bioinformatics from execution to validation. It also argues that this shift will magnify the existing problems of Western bias in genomics, and proposes some remedies.

There are many good things to say about this perspective! The focus on validation is very appropriate, and well suited to the audience of Cell Genomics. The topic is also "future proofed" in the sense that while the particulars of software and approach will undoubtedly change, the fundamental issue is here to stay. The definition of agentic genomics is also well thought out, reasonably precise, and distinguishes it from other related concepts.

Our opinion is that this is a potentially important paper and it should be published. It was very generative to read and discuss.

However... there are a number things to be critical about.

The discussion and presentation of the software platforms was largely superficial and seemed to be pretty focused on BioClaw. We sympathize with the authors on the conundrum of how to present the concepts without specifics, but right now the balance seems a bit off. BioClaw is sort of discussed, but not really; other packages are mentioned, but not described; etc. The Acknowledgements implies that BioClaw was strongly involved in this paper.

"Validation" is an overloaded term that is used in many different ways.

e.g. the Research grade validation in Table 2 is focused on technical correctness, and is quite a different beast from the other kinds of validation mentioned. Scientific validation through peer review and external experimentation is also part of the lifecycle and should be mentioned.

We did not see any mentions of "circular tuning" as a danger - the situation where independent benchmarks are used to fine tune agentic workflows, so that the benchmarks are no longer independent. In computer science this is normal (which is not good!) and it will cause serious problems in biology if it is adopted.

The idea that agentic workflows could lead to robust and widspread p-hacking is implied but should probably be mentioned.

Page 8 - Talks about different failure mechanisms for different agentic tools out there:

These mechanisms operate at different layers (execution, reasoning, governance) and are not directly comparable as alternative solutions.

Automated self-repair -> addresses syntactic or runtime errors
Multi-agent self-reflection -> reasoning
Community-driven -> governance

The use of "SKILL" as a term of art seems BioClaw specific. "Tools", "actions", and others are similar concepts. Skill is not a universal standard as implied in this paper.

More generally, the paper raised some significant conceptual issues for us. It's not clear how many of these can reasonably be addressed,

but we feel that some discussion of some of them would improve the paper.

* increasing ease of hypothesis investigation suggests that we need to improve our hypothesis generation approach. But this (the "lifecycle" and genesis of hypotheses) is not really mentioned in the paper.

* one of the main uses of agentic workflows could be in validating and exploring results during publication review. Worth mentioning.

* biology is full of both known unknowns (discussed to some degree in this paper) and unknown unknowns. This latter category especially causes problems for overly confident AI/DL approaches. What kinds of mitigations could be introduced?

* it is interesting to speculate about an increased need for/interest in/ market for/ecosystem of tools that do validation for known unknowns, e.g. "unexpected allele frequency" distributions or the like. Right now this is handled by people looking at results and using experience to evaluate, but maybe this could be partially encoded in software?

* how will agentic workflows interact with publication? Will there/could there be ways to publish workflows, freeze workflows for reuse and provenance in adaptation, etc?

* have the authors thought about how study preregistration could engage with agentic workflows? Seems like a natural fit.

Even more generally, this paper and the topic of agentic workflows overall is enmeshed in the dysfunction of our current scientific culture: there are fundamental problems and issues around open data,

reproducibility, p-hacking, peer review, and so on that will impact and be impacted by agentic workflows, and this should at least be mentioned.

Minor issues -

Box 1 is not referenced anywhere in the text. Also, did this use BioClaw?

More generally, no sufficient methodological detail for either Box 1 or Table 1. No mention of models used, agents framework, protocol, metrics or what was the output. Nothing quantifiable.

the citations after "multiple independent systems" (9, 15, 19, 20) are improperly formatted.

Some reference to "out of distribution" errors might tie the issue of ethics and validation to appropriate CS literature.

References needed for the claims made in "Bias detection during execution."

Citations for GA4GH and FAIR are missing.

Failure handling in CellArtia is missing from the paragraph "These systems address..."

The FDA regulatory framework should be referenced in Table 2.

Reviewer 2:

The manuscript defines "agentic genomics" as LLM-powered autonomous agents that select tools, execute, and self-correct using a domain-constrained skill

library. Authors argues that the bottleneck has shifted from pipeline construction to validation, recasting the researcher from producer to judge. Authors survey four systems—CellAtria (scRNA-seq), AutoBA (multi-omic), Bio-Copilot (multi-agent), and ClawBio (first author's own community skill library)—then propose a three-tier validation framework with escalating QC, audit, and regulatory requirements plus deterministic replay.

Som eissues I found:

The main issue is that this manuscript reads more like a "white paper" or a "mission statemn" rather than an actual research paper.

Table 1 is an asssertion: "Values reflect convergent observations… Formal benchmarking… remains a priority."

The perturbation test was never actually run on any of the four systems (or did this reviewer missed that)

Three tiers proposed (Table 2); none of the four systems is classified or audited against them. A framework that the authors don't apply to their own ecosystem survey is hard to take as actionable.

Why these four? BioMANIA, GeneGPT, BioCoder, Biomni, GeneAgent, STAR-style frameworks are absent.

Authors' response to the first round of review

Response to the Editor and Reviewers

Manuscript: CELL-GENOMICS-D-26-00316 **Title:** Agentic Genomics: From Pipeline Automation to Autonomous Validation **Authors:** Manuel Corpas, Segun Fatumo, Heinner Guio **Corresponding author:** Manuel Corpas (m.corpas@westminster.ac.uk) **Date:** 3 June 2026

Dear Dr Nicholson,

Thank you for the opportunity to revise our Perspective, and please pass our thanks to both reviewers. The comments were constructive and have materially strengthened the paper. We have addressed every point. References to sections below are to the revised manuscript; new references are numbered 24 to 36 and were each verified against the primary source.

Three structural changes summarise the revision. First, we now apply our own validation framework to the systems we survey: a new Table 3 classifies CellAtria, AutoBA, Bio-Copilot, and ClawBio against the three tiers, using only their published validation evidence. Second, we have reduced the prominence of ClawBio so that it reads as one example among peers rather than the centre of the paper. Third, we have sharpened the validation discussion to distinguish technical from scientific validation and to address circular benchmark tuning, out-of-distribution inputs, agent-mediated p-hacking, preregistration, and the entanglement of agentic genomics with broader scientific culture.

We have kept the article in the Perspective register, in line with your guidance that a substantial shift away from that tone is not required. We note explicitly in the Summary that this is a Perspective rather than a benchmarking study. We have selected transparent peer review and are content for the full exchange to be published. The revision adds a STAR Methods section, a data and code availability statement, and a key resources table, and it documents the one computational result we actually ran, a live perturbation test on the current ClawBio release.

We hope you find the revision suitable for publication in Cell Genomics.

Yours sincerely, Manuel Corpas, on behalf of all authors

Reviewer 1

> "We did not see any mentions of 'circular tuning' as a danger, the situation where independent benchmarks are used to fine tune agentic workflows, so that the benchmarks are no longer independent. In computer science this is normal (which is not good!) and it will cause serious problems in biology if it is adopted."

Response. We agree, and we have added a dedicated paragraph, *Avoiding circular benchmark tuning*, to the *A tiered validation framework* section. It describes how benchmarks intended as independent evaluations are progressively absorbed into the systems they are meant to test, names the genomics benchmarks most exposed to this (Genome in a Bottle, curated scRNA-seq atlases, CPIC guidelines), and proposes two structural protections: held-out partitions released only at evaluation time, and periodic benchmark rotation. New references: Recht et al. 2019 (ref 31) and Nosek et al. 2018 (ref 32).

> "The idea that agentic workflows could lead to robust and widespread p-hacking is implied but should probably be mentioned."

Response. We have added a bolded *Challenges* item, *Agent-mediated p-hacking and exploration inflation*, stating the multiple-comparisons argument directly: the cost of running each analytical variant collapses while the multiple-testing burden does not. We also added a forward reference to it from the *Accelerated hypothesis exploration* item in Opportunities so that it is easy to find. New reference: Head et al. 2015 (ref 33).

> "These mechanisms operate at different layers (execution, reasoning, governance) and are not directly comparable as alternative solutions. Automated self-repair addresses syntactic or runtime errors; Multi-agent self-reflection addresses reasoning; Community-driven addresses governance."

Response. We have rewritten the offending paragraph in *An emerging ecosystem*. AutoBA's automated repair is now framed as an execution-layer mechanism, Bio-Copilot's self-reflection as reasoning-layer, and ClawBio's community remediation as governance-layer, with CellAtria added as a design-layer preventive posture. We state explicitly that these are complementary layers rather than competing solutions, and we removed the unsupported claim that their convergence is itself evidence of a structural shift.

> "The use of 'SKILL' as a term of art seems BioClaw specific. 'Tools', 'actions', and others are similar concepts. Skill is not a universal standard as implied in this paper."

Response. We have added a sentence to *Agent-native skill libraries: the critical interface* acknowledging that "tools", "actions", and "skills" are convergent terms across ecosystems, and stating that we use "skill" for definiteness, not to assert a universal standard.

> "Increasing ease of hypothesis investigation suggests that we need to improve our hypothesis generation approach. But this (the 'lifecycle' and genesis of hypotheses) is not really mentioned in the paper."

Response. We have added an *Opportunities* item, *Hypothesis generation becomes the new bottleneck*, and a sentence in the validation section noting that, across the research lifecycle, the locus of rigour shifts upstream to hypothesis generation, framing, and preregistration as the cost of testing collapses.

> "One of the main uses of agentic workflows could be in validating and exploring results during publication review. Worth mentioning." And: "How will agentic workflows interact with publication? Will there/could there be ways to publish workflows, freeze workflows for reuse and provenance in adaptation, etc?"

Response. Both points are addressed in a new *Opportunities* item, *Publishable workflows and agent-assisted peer review*, covering versioned, citable workflow artefacts deposited alongside manuscripts and agent-assisted re-execution by reviewers. We also flag reviewer-side validation as a use case when introducing the tiered framework.

> "Biology is full of both known unknowns and unknown unknowns. This latter category especially causes problems for overly confident AI/DL approaches. What kinds of mitigations could be introduced?" And: "It is interesting to speculate about an increased need for tools that do validation for known unknowns, e.g. 'unexpected allele frequency' distributions or the like."

Response. We have added a paragraph, *Out-of-distribution inputs and unknown unknowns*, to the validation section. It proposes out-of-distribution detectors as first-class skill components (population mismatch, sequencing-platform drift, tissue-context mismatch) and names the available machinery (calibration curves, conformal prediction, density-ratio estimators). The reviewer's "unexpected allele frequency" example is used directly here and in the *Bias detection during execution* item. New reference: Hendrycks and Gimpel 2017 (ref 30).

> "Have the authors thought about how study preregistration could engage with agentic workflows? Seems like a natural fit."

Response. We have added an *Opportunities* item, *Preregistration becomes operationally lightweight*, showing how a preregistered plan can be expressed as a fixed sequence of skill invocations and how deviations become detectable from execution traces (ref 32).

> "This paper and the topic of agentic workflows overall is enmeshed in the dysfunction of our current scientific culture: there are fundamental problems and issues around open data, reproducibility, p-hacking, peer review, and so on. This should at least be mentioned."

Response. We have added a *Challenges* item, *Entanglement with the broader scientific culture*, stating that agentic genomics inherits and may amplify these problems and cannot succeed in isolation from broader reform.

> "Validation is an overloaded term. Research-grade validation in Table 2 is focused on technical correctness, and is quite a different beast from the other kinds of validation mentioned. Scientific validation through peer review and external experimentation is also part of the lifecycle and should be mentioned."

Response. The validation section now opens with a one-sentence definition: we use

distinct from scientific validation through peer review, external experimentation, and independent replication, which the framework does not displace. We add that agentic genomics heightens rather than reduces the demand on the latter.

Reviewer 1, minor points

> "Box 1 is not referenced anywhere in the text. Also, did this use BioClaw? More generally, no sufficient methodological detail for either Box 1 or Table 1. No mention of models used, agents framework, protocol, metrics or what was the output. Nothing quantifiable."

Response. Box 1 is now referenced in the main text (*The agentic shift*). On reflection we judged that the honest way to meet this comment was not to dress Box 1 up as a logged experiment but to present it as what it is. Box 1 is now an explicitly illustrative example: it reports no timings, counts, or comparative measurements, and the STAR Methods states that no systematic benchmark was conducted for this Perspective and names the tools the agent orchestrates. Table 1 is likewise reframed (see Reviewer 2 below). The one computational result we did run, the perturbation demonstration, is reported with its date, inputs, and outcome in the STAR Methods.

> "The citations after 'multiple independent systems' (9, 15, 19, 20) are improperly formatted."

Response. These citations now appear in the Table 1 note and are formatted consistently; final styling will follow Cell Press conventions at production.

> "Some reference to 'out of distribution' errors might tie the issue of ethics and validation to appropriate CS literature."

Response. Done, via Hendrycks and Gimpel 2017 (ref 30) in the new out-of-distribution paragraph.

> "References needed for the claims made in 'Bias detection during execution.'"

Response. The *Bias detection during execution* item now cites Martin et al. 2019 (ref 36) for the documented miscalibration of polygenic risk scores across ancestries.

> "Citations for GA4GH and FAIR are missing."

Response. GA4GH is now cited (ref 11) and FAIR is cited with the canonical Wilkinson et al. 2016 reference (ref 34), both in *Agent-native skill libraries*.

> "Failure handling in CellAtria is missing from the paragraph 'These systems address…'"

Response. The rewritten layering paragraph now includes CellAtria as a design-layer, preventive posture alongside the execution, reasoning, and governance exemplars.

> "The FDA regulatory framework should be referenced in Table 2."

Response. The clinical-grade tier of Table 2 and the clinical-safety item in Challenges now cite Gerke et al. 2020 (ref 35) on regulating AI/ML-based software as a medical device.

Reviewer 2

> "The main issue is that this manuscript reads more like a 'white paper' or a 'mission statement' rather than an actual research paper."

Response. We note the editor's guidance that the Perspective tone is appropriate, and we have added an explicit statement to the Summary that this is a Perspective rather than a benchmarking study. Within that register we have increased rigour at each of the specific points the reviewer raised below, so that every claim is either evidenced or labelled as qualitative.

> "Table 1 is an assertion: 'Values reflect convergent observations… Formal benchmarking… remains a priority.'"

Response. Agreed. Table 1's setup-time row is now expressed as qualitative bands (hours for manual workflows versus minutes for agent-mediated ones) rather than numeric ranges, and its note states that no cell contains a measured value and that every entry is a categorical characterisation of typical behaviour.

> "The perturbation test was never actually run on any of the four systems (or did this reviewer miss that)."

Response. It had not been; it has now. In *The validation bottleneck* we report a live re-run of the empty-input perturbation on the current ClawBio release. Presented with empty, malformed, and content-free genotype files, the pharmacogenomic skill halts with an explicit file-format error and a non-zero exit status in every case, rather than the silent "all normal" report that the original community audit exposed. The run is documented in the STAR Methods with its date and inputs. We are explicit that this is a skill-level input-perturbation demonstration, not a multi-system benchmark, and that ClawBio is used only as one openly inspectable instance of a general pattern.

> "Three tiers proposed (Table 2); none of the four systems is classified or audited against them. A framework that the authors don't apply to their own ecosystem survey is hard to take as actionable."

Response. This was the most important comment, and we have acted on it directly. The new Table 3 classifies all four surveyed systems against the framework using only their published validation evidence, placing each at the highest tier for which evidence exists. The conservative result, that all four currently sit at research-grade with partial movement towards benchmarked and none at clinical-grade, is reported as a finding about the maturity of the field. We also state that these placements are provisional and rest solely on publicly available evidence.

> "Why these four? BioMANIA, GeneGPT, BioCoder, Biomni, GeneAgent, STAR-style frameworks are absent."

Response. We have added a paragraph to *An emerging ecosystem* positioning each named system against our four-condition definition: BioCoder (ref 24) is a benchmark rather than an executor; GeneGPT (ref 25) is a retrieval copilot; GeneAgent (ref 26) lacks a curated skill library; BioMANIA (ref 27) sits at the boundary of the definition; and Biomni (ref 28) is a general-biomedical agent that appeared while this Perspective was being prepared. STAR-style and other frameworks adapted from the general computer-science literature are acknowledged via the two existing systematic reviews (refs 12 and 13).

Referees' report, second round of review

Reviewer 1:

Thank you for addressing our concerns!

Authors' response to the second round of review
