# Supplementary material for: Agentic genomics: From pipeline automation to autonomous validation
Source: Cell Genom. 2026 Jul 21;6(8):101305. doi: 10.1016/j.xgen.2026.101305 (PMC13477014; doi:10.1016/j.xgen.2026.101305)
Supplement: Supplementary file 3 [file mmc3.pdf]

Cell Genomics

CellPress
OPEN ACCESS

Perspective

Agentic genomics: From pipeline automation to autonomous validation

Manuel Corpas,[1,*] Heinner Guio,[2] and Segun Fatumo[3,4,*]
[1]School of Life Sciences, University of Westminster, London, UK
[2]Centro de Investigación en Bioingeniería (BIO), Universidad de Ingeniería y Tecnología (UTEC), Lima, Perú
[3]MRC/UVRI and LSHTM Uganda Research Unit, Entebbe, Uganda
[4]Precision Healthcare University Research Institute, Queen Mary University of London, London, UK
*Correspondence: m.corpas@westminster.ac.uk (M.C.), s.fatumo@qmul.ac.uk (S.F.)
https://doi.org/10.1016/j.xgen.2026.101305

SUMMARY

Genomics has entered a phase in which AI agents can autonomously discover, configure, execute, and chain bioinformatics operations from natural-language instructions. We term this paradigm "agentic genomics": the delegation of multi-step genomic analyses to autonomous software agents that select tools, manage dependencies, and adapt execution in response to intermediate results, mediated by large language models (LLMs) and constrained by domain-specific skill libraries. We argue that agentic genomics shifts the bottleneck in computational biology from pipeline construction to validation. We examine emerging systems, including CellAtria, AutoBA, Bio-Copilot, and ClawBio, and assess their divergent architectures. We propose a tiered validation framework spanning research-grade, benchmarked, and clinical-grade analyses and argue that equity-aware design must be a systems requirement rather than an optional aspiration. We identify the infrastructure needed to make agentic genomics trustworthy.

DEFINING AGENTIC GENOMICS

We define agentic genomics as follows: agentic genomics is the use of autonomous AI agents, powered by large language models (LLMs) and operating within domain-constrained skill libraries, to discover, plan, execute, and iteratively refine multi-step genomic analyses, where the agent exercises runtime decision-making over tool selection, parameterization, error handling, and output evaluation.

This definition establishes four necessary conditions. The first is autonomy: the agent must make decisions during execution, not merely follow a static workflow specification. The second is domain constraint: the agent must operate within a structured library of validated operations (skills), not generate code *ad hoc* from a general-purpose model. The third is iterative refinement: the agent must be capable of evaluating intermediate results and modifying its approach accordingly, including error diagnosis and self-repair. The fourth is natural-language mediation: the interface between the researcher and the analysis must be natural language, enabling researchers without programming expertise to direct complex analyses.

These conditions are jointly necessary. A system that satisfies only a subset falls into one of several existing categories, and conflating these categories with agentic genomics obscures the genuinely novel properties of the paradigm. Critically, the definition is empirically testable: a system fails to qualify as agentic if, when presented with identical input under perturbed intermediate outputs (e.g., a tool returning an unexpected error or an anomalous result), it does not alter its execution strategy. This perturbation test distinguishes genuine runtime decision-making from pre-specified branching logic; we provide a worked example of the test applied to one system in the section the validation bottleneck: the new rate limiting step below.

What agentic genomics is not

It is not workflow automation

Nextflow,[1] Snakemake,[2] and Galaxy[3] provide powerful, reproducible pipeline orchestration, but their workflows are specified in advance by a human developer. The workflow manager executes a fixed directed acyclic graph (DAG); it does not decide at runtime which tools to use, nor does it adapt its strategy when intermediate results are unexpected. Agentic genomics builds on workflow infrastructure (an agent may invoke a Nextflow pipeline as one step in a larger analysis) but is not reducible to it.

It is not AutoML or automated pipeline selection

Systems that search over a predefined space of model architectures or hyperparameters operate within a fixed search space defined by a human engineer. They optimize within constraints; they do not formulate the analysis strategy itself.

It is not LLM-assisted scripting

Using a chatbot to generate a Python script for variant filtering is useful but does not constitute agentic genomics. The LLM produces code; the human executes and evaluates it. There is no autonomous execution, no runtime adaptation, and no domain-constrained skill library. This mode of interaction, sometimes called "vibe coding,"[4] is a precursor to the agentic paradigm but lacks its defining properties.

© 2026 The Authors. Published by Elsevier Inc.
This is an open access article under the CC BY license (http://creativecommons.org/licenses/by/4.0/).

It is not a general-purpose AI copilot

Biomedical AI copilots that answer clinical questions or summarize literature operate as information retrieval systems. They do not execute multi-step computational analyses against real data.

The distinguishing feature of agentic genomics is that the agent acts: it executes tools, reads their output, decides what to do next, and delivers results. The researcher's role shifts from constructing the analysis to evaluating it. This shift, from production to judgment, is the central consequence of the paradigm.

THE COMPUTATIONAL BURDEN THAT CREATED THE OPPORTUNITY

Biology has become a data-saturated science. The sequencing of a single human genome produces approximately three billion base pairs of information, and the clinical interpretation of that genome requires alignment, variant calling, functional annotation, literature cross-referencing, and synthesis into actionable reports. Each step depends on specialized software with its own dependencies, version histories, and configuration requirements.[1,2]

A typical genomic analysis pipeline chains together dozens of tools, each with parameters that must be tuned to the specific data type, sequencing platform, and biological question. Workflow managers such as Nextflow, Snakemake, and Galaxy emerged precisely because manual orchestration is unsustainable.[3] Yet even with these frameworks, the barrier to entry for researchers without formal computational training remains high. A biologist who wants to analyze their own sequencing data must either learn to code, hire someone who can, or rely on graphical interfaces that may not support the analysis they need.

This tension between the ubiquity of genomic data and the scarcity of computational expertise has defined the field for two decades. Modern LLMs have begun to dissolve that barrier, not by teaching researchers to code but by making code generation, execution, and debugging available through natural language.

THE AGENTIC SHIFT

The first wave of LLM adoption in the life sciences focused on information retrieval: summarizing papers, answering questions about pathways, and extracting structured data from text.[5–7] This was useful but incremental.

The second wave is qualitatively different. Modern LLMs can write, debug, and execute code. When connected to file systems, databases, and command-line tools, they become autonomous agents that plan multi-step operations, execute them, and adapt based on intermediate results.[8,9] Recent systematic reviews have cataloged dozens of such systems across genomics, proteomics, spatial biology, and biomedicine,[10–12] confirming that LLM-based agents have moved from isolated prompts to coordinating multi-tool pipelines with iterative decision-making.

In genomics, a researcher can now describe an analysis in natural language and have an agent construct and execute the corresponding pipeline. The agent identifies required tools, downloads reference data, configures parameters, runs the analysis, and returns results (Box 1).

Box 1. Illustrative example: Agent-mediated exome analysis

To make the agentic workflow concrete, we describe, illustratively, how an AI agent on a local workstation handles a trio whole-exome analysis. This is a qualitative illustration of the pattern, not a benchmark: no timings, counts, or comparative measurements are reported. Task: identify candidate pathogenic variants from a trio whole-exome sequencing dataset (proband and parents) and flag clinically relevant findings.

Agent execution:

1. The agent received natural-language instruction: "Run a trio exome analysis on these three FASTQ files. Identify *de novo* and compound heterozygous variants. Flag clinically relevant findings."
2. The agent selected the tools: fastp (adapter trimming and QC), BWA-MEM2 (alignment to GRCh38), GATK HaplotypeCaller (variant calling), DeepVariant (parallel variant calling for cross-validation), and SnpEff + ClinVar (annotation).
3. During execution, BWA-MEM2 flagged an incompatible index format. The agent diagnosed the error, rebuilt the index, and resumed alignment without human intervention. This step is a concrete instance of the perturbation test in the definition above: a perturbed intermediate result altered the agent's execution strategy at runtime.
4. The agent applied trio-aware filtering (Mendelian violation check and *de novo* probability scoring), annotated against ClinVar and OMIM, and generated a ranked candidate list.
5. The agent produces a ranked list of candidate variants, including *de novo* and compound heterozygous candidates with ClinVar classifications and supporting evidence, which a human reviewer then evaluates.

Key observation: the agent's value here is not speed but autonomous error recovery (step 3) and systematic cross-validation (running two variant callers in parallel), which a time-pressured analyst might omit. The corresponding risk is also illustrative: an agent may fail to flag a borderline quality-control metric that an experienced analyst would notice and investigate. This trade-off, faster and more systematic execution set against the loss of a human's intuitive skepticism, is the pattern this perspective examines. Further details on the tools and the illustrative nature of this example are given in the methods and supplemental information.

To ground this claim, Table 1 provides a structured qualitative comparison of human-directed and agent-mediated execution across representative genomic workflows.

AGENT-NATIVE SKILL LIBRARIES: THE CRITICAL INTERFACE

The power of agentic genomics scales with the quality of the operations agents can perform. A general-purpose LLM can generate bioinformatics code, but the output varies between sessions, may contain subtle errors, and lacks the specificity that domain experts build into workflows over years of practice.

This problem has a structural solution: agent-native skill libraries. A skill is a self-contained, versioned unit of bioinformatics functionality that encapsulates code, configuration, data

CellPress
OPEN ACCESS

Table 1. Qualitative comparison: Human-directed vs. agent-mediated genomic workflows

Metric	Exome analysis (human)	Exome analysis (agent)	scRNA-seq (human)	scRNA-seq (agent, CellAtria[13])
Setup time (tool selection, configuration)	hours	minutes	hours	minutes
Execution monitoring required	continuous	minimal (agent handles errors)	continuous	minimal
Reproducibility	variable (depends on documentation)	high (skill specification fixed)	variable	high (standardised pipeline)
Error recovery	manual debugging	automated diagnosis and repair	manual	automated with fallback
Prerequisite expertise	bioinformatics training	domain knowledge for validation	bioinformatics + statistics	domain knowledge for validation
Primary failure mode	configuration errors, version conflicts	silent plausible-looking errors	parameter tuning errors	hallucinated annotations, missed QC

This table is a qualitative synthesis, not a benchmark. No cell contains a measured value. Every entry is a categorical characterization of typical behavior: the setup time row contrasts the order of magnitude (hours for manual workflows vs. minutes for agent-mediated ones), consistent with reports from early adopters of multiple independent systems,[13–16] and the remaining rows are qualitative. We include the table to convey the direction of the shift, not to assert measured performance. Formal benchmarking across standardized datasets, with confidence intervals and controlled multi-site evaluation, remains a priority for the field and is one of the infrastructure gaps this perspective identifies.

references, input/output specifications, and test suites. Skills are designed to be discovered and executed by AI agents through semantic search and natural-language invocation. They are the bioinformatics equivalent of a well-documented API, with the critical addition of a natural-language specification (typically a SKILL.md file) that enables an agent to discover the right skill for a given question, understand its requirements, and invoke it without human configuration. Different ecosystems use convergent but non-identical terms for this concept: "tools" in the Anthropic and OpenAI function-calling literatures, "actions" in some autonomous-agent frameworks, and "skills" in ClawBio and several adjacent projects. We use "skill" throughout this perspective for definiteness, not to assert a universal standard; the structural argument applies equally to any unit that encapsulates a validated, semantically discoverable, agent-invokable operation.

The concept of modular, reusable bioinformatics components is not new. Galaxy tools, Nextflow modules, nf-core pipelines,[1] and CWL/WDL workflow definitions have long provided structured ways to package analyses. What agent-native skills add is the semantic layer: the skill encapsulates the domain expert's decisions, including the rationale for parameter choices, expected failure modes, and test data for verification. Skills complement rather than replace existing workflow infrastructure; a skill can wrap a Nextflow module or invoke Galaxy tools as part of its execution. This interoperability with established standards, including GA4GH data-sharing frameworks[17] and FAIR principles for computational biology,[18] is essential: the agentic paradigm must build on, not displace, two decades of community investment in reproducible infrastructure.

AN EMERGING ECOSYSTEM

Several independent groups have converged on architectures that share the core properties of agentic genomics, each emphasizing different aspects of the paradigm.

CellAtria,[13] developed at AstraZeneca, provides an agentic framework for single-cell RNA sequencing (scRNA-seq) that enables dialogue-driven, document-to-analysis automation. A researcher can point the agent at a published paper; CellAtria extracts metadata, retrieves the corresponding dataset from public repositories, and executes a standardized scRNA-seq pipeline through a chatbot interface. CellAtria adopted an LLM-mediated tool-centric paradigm rather than *ad hoc* code generation, explicitly prioritizing reproducibility over flexibility.

AutoBA[14] takes a complementary approach, providing an autonomous agent for fully automated multi-omic analyses. Users supply minimal input (a data path, a description, and an objective), and AutoBA proposes analysis plans, generates and executes code, and returns results. Its automated code repair mechanism addresses the silent-failure problem: when execution fails, the agent diagnoses and corrects its own code, reducing the need for human debugging.

Bio-Copilot[19] extends the multi-agent paradigm, deploying specialized agent groups that collaborate on complex bioinformatics tasks. Its architecture incorporates self-reflection protocols, a shared knowledge database, and structured human-agent collaboration, achieving higher completion rates and fewer correction rounds than general-purpose LLMs on standardized omics workflows.

ClawBio[15] emphasizes community-driven skill curation. Within weeks of its public release, wet-lab biologists with limited coding experience were contributing functional, tested skills to the library, using LLMs as the programming intermediary, an early signal that domain experts can encode analytical knowledge into agent-executable modules without becoming software engineers.

Our four-condition definition narrows the field, and several related systems are worth positioning against it. BioCoder[20] is a benchmark for evaluating LLM code generation in bioinformatics rather than a system that executes analyses on user data. GeneGPT[21] augments LLMs with NCBI web APIs to retrieve

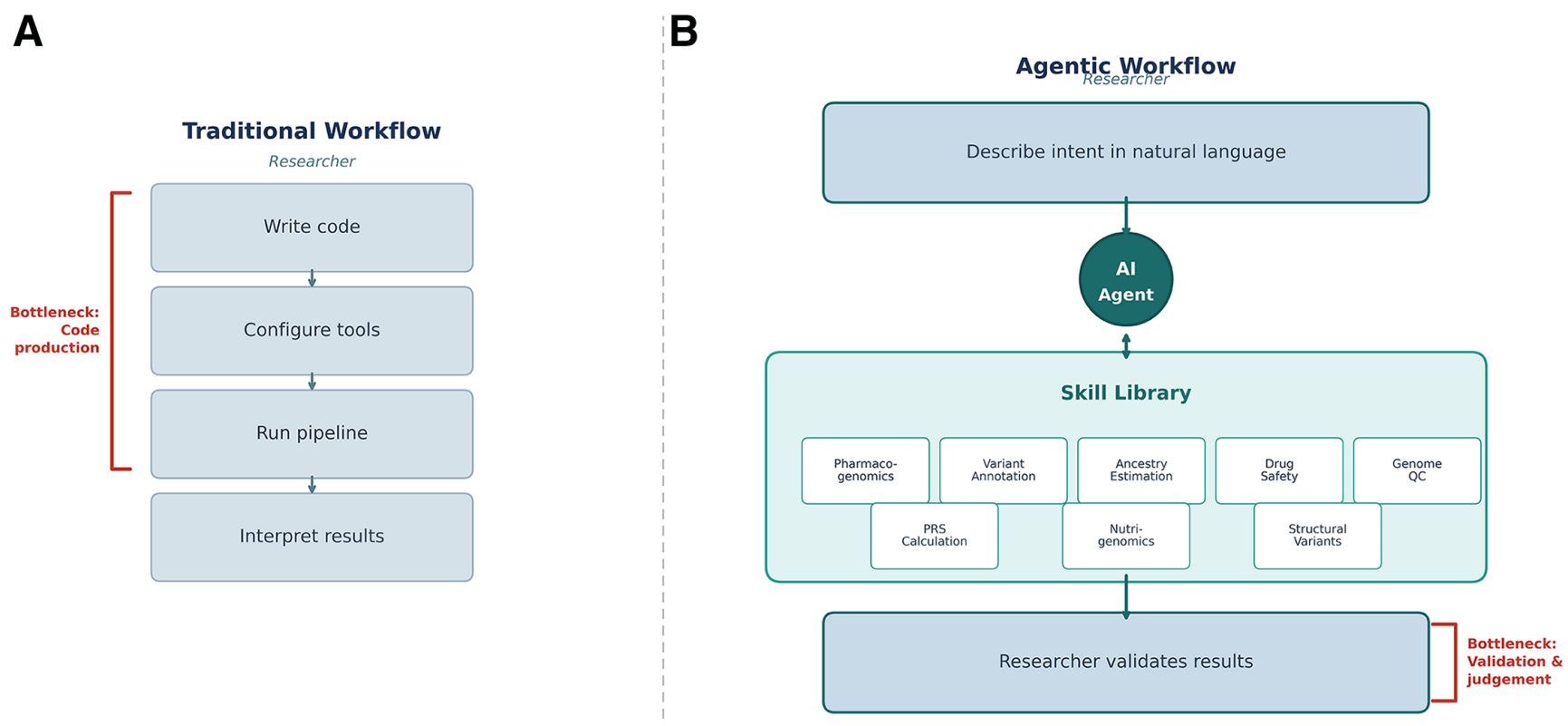

Figure 1. The agentic genomics paradigm
(A) Traditional genomics workflow, where the researcher writes code, configures tools, runs pipelines, and interprets results; the bottleneck is code production.
(B) Agentic workflow, where the researcher describes intent in natural language, an AI agent discovers and executes skills from a modular library, and the researcher validates results; the bottleneck shifts to validation and judgment.

biomedical information; it falls within the general-purpose copilot category excluded earlier in this perspective. GeneAgent[22] performs autonomous gene-set analysis with database-grounded self-verification but operates without a curated, versioned, agent-discoverable skill library and is closer in spirit to a domain-specific question-answering agent than to a multi-step analytical executor. BioMANIA[23] converts natural-language queries into invocations of documented Python libraries; its skill surface is the implicit API of the underlying library rather than an explicit, versioned, tested specification, placing it at the boundary of our definition rather than squarely within it. Biomni,[24] released as this perspective was being prepared, presents a structured action space across biomedical subfields and satisfies the structural conditions of the paradigm at the broader biomedical layer rather than genomics specifically; we expect general-purpose biomedical agents of this kind to converge with the genomics-specific systems surveyed here as the field matures. The wider landscape of LLM-augmented biomedical tools, including autonomous agent frameworks adapted from the general computer science literature, is reviewed in detail elsewhere.[10,11]

These systems address failure at different layers of the agent stack and are best understood as complementary rather than as alternative solutions to a single problem. AutoBA's automated code repair operates at the execution layer, catching and resolving syntactic or runtime errors before they propagate. Bio-Copilot's multi-agent self-reflection operates at the reasoning layer, surfacing logical inconsistencies between agents before plans reach execution. ClawBio's community-driven adversarial testing and transparent remediation operate at the governance layer, providing the institutional process by which failures are surfaced, audited, and corrected across the user base. CellAtria sits earlier still, at the design layer: by constraining analyses to a standardized, reproducibility-first pipeline, it prevents whole classes of failure from arising rather than catching them after execution. A mature agentic ecosystem will likely need all of these layers, with comparative evaluation tracking which failure modes each layer reliably catches and which fall through the cracks between them.

This model inverts the traditional relationship between bioinformaticians and biologists. In the conventional workflow, biologists bring the questions and bioinformaticians write the code. In the agentic model, biologists can both formulate the questions and encode the answers because the coding step is mediated by AI. The bioinformatician's irreplaceable contribution shifts from writing software to validating it (Figure 1).

THE VALIDATION BOTTLENECK: THE NEW RATE-LIMITING STEP

The most consequential challenge in agentic genomics is not generating analyses but verifying them. AI agents can produce results at a rate that far exceeds a human researcher's capacity for review. This creates a new class of risk: high-throughput, low-validation science.

Throughout this perspective, we use the word "validation" in a specific sense: technical and methodological checks on agentic analyses. This is distinct from scientific validation through peer review, external experimentation, and independent replication, which remains the mature mechanism by which findings are accepted into the scientific record and which the tiered framework introduced below does not displace. Agentic genomics changes the technical bottleneck; it heightens, rather than reduces, the demand on the scientific one, as the volume of analyses produced now exceeds what conventional peer review was designed to absorb. Seen across the research life cycle, this shifts the locus of rigor upstream as much as downstream: as the cost of hypothesis testing collapses, hypothesis generation, careful framing, and preregistration become the activities that most determine whether the paradigm yields reliable knowledge.

The risk is not hypothetical. Failure modes are consistent across independently developed systems. AutoBA evaluations revealed cases where autonomously generated pipelines omitted critical steps or selected inappropriate tools for the data type.[14] A randomized clinical trial of GPT-4-augmented diagnostic reasoning found no significant improvement over conventional resources (median: 76% vs. 74%, $p = 0.60$), even though the model alone scored substantially higher than physicians[25]; the gap between capability and outcome is the validation gap. Reviews of single-cell agentic systems have noted incomplete experimental designs and inconsistent tool recommendations for identical queries.[12] Boiko et al. documented cases where autonomous agents produced syntactically correct but scientifically invalid experimental protocols that passed

basic execution checks.[9] In the early weeks of ClawBio, a computational biologist audited the library and discovered that one skill, when presented with an empty input file containing no genomic data, silently returned "all-normal" results, including recommended dosages for 51 drugs.[15] The pattern is consistent across systems: silent degradation to plausible-looking but incorrect results.

We re-ran this perturbation on the current ClawBio release as a worked example of the perturbation test defined above. When presented with empty, malformed, and content-free genotype files, the pharmacogenomic skill now halts with an explicit file-format error and a non-zero exit status in every case rather than returning a report. The silent-failure path that the original audit exposed has been closed by the governance-layer remediation process: community audit, public disclosure, and a fix that converts a silent degradation into a loud, diagnosable failure. We use ClawBio here only as one concrete, openly inspectable instance of a pattern that is not specific to it: AutoBA's execution-layer code repair and Bio-Copilot's reasoning-layer self-reflection are analogous corrective mechanisms sited at different layers of the stack. This is the behavior the perturbation test is meant to elicit, and it illustrates why the governance layer is a first-class component of validation rather than an afterthought.

This class of failure, where the absence of data or flawed logic silently degrades into a plausible-looking result, is not unique to agentic software. It occurs in traditionally developed bioinformatics tools as well. But the speed of AI-mediated development amplifies the risk because skills and pipelines can be written, shared, and deployed faster than they can be audited.

Out-of-distribution inputs and unknown unknowns

Silent degradation has a known-unknown counterpart that current agentic systems handle poorly: out-of-distribution inputs, where the data the agent is processing differ systematically from the distribution on which the underlying tools and models were validated.[26] A variant caller calibrated on short-read benchmarks applied to long-read sequencing data is operating out of distribution; a cell-type classifier trained on healthy reference atlases applied to a diseased tissue is operating out of distribution; an allele frequency filter calibrated on aggregate population databases applied to a cohort whose ancestry composition differs from the calibration set is operating out of distribution. The danger is that current agents will execute these analyses to completion and return results without flagging the distributional shift. Genuinely robust agentic systems must include out-of-distribution detectors as first-class skill components: tests for population mismatch, sequencing-platform drift, tissue-context mismatch, and other forms of train-test divergence that experienced human analysts often catch by intuition. The community already has the conceptual machinery, including calibration curves, conformal prediction intervals, and density-ratio estimators[26]; what is missing is its routine integration into agentic pipelines.

The hallucination problem compounds these risks. LLM hallucination, the generation of plausible but factually incorrect output, is well documented.[27] In genomics, hallucination takes particularly insidious forms: an agent might cite a non-existent gene-disease association, fabricate a reference, or generate a variant annotation that conflates unrelated genetic loci. Clinical LLM benchmarks have shown that even models fine-tuned for medical applications produce confident but incorrect outputs on biomedical questions.[28] The jagged capability profile of current LLMs, performing at an expert level on some tasks while failing at elementary ones, often without clear signals about which mode they are operating in, makes this risk especially difficult to manage.

This creates a paradox at the heart of the democratization argument. Agentic genomics lowers the barrier to generating analyses, but it does not lower the barrier to evaluating them. The skills required for evaluation, deep domain knowledge, familiarity with common failure modes, and intuition about plausible results are built over years and cannot be shortcut by AI. A researcher with 20 years of experience in variant interpretation will recognize when allele frequencies are implausible, when gene-disease associations contradict established biology, and when a filtering step has been silently dropped. A novice user will not. The democratization is real but partial. It expands the capacity to produce; it does not expand the capacity to judge.

A TIERED VALIDATION FRAMEWORK

The validation challenge requires a structured response. We propose a three-tier validation framework that calibrates scrutiny to consequence (Table 2). The framework governs developer- and author-side validation; the same skill-level infrastructure also supports reviewer-side validation, a use case we return to under opportunities.

This framework addresses a specific regulatory gap. For CLIA/CAP-certified clinical laboratories, if an agent selects a variant-calling pipeline based on a natural-language instruction, the laboratory must be able to lock down version control, validate the selected configuration, and maintain an audit trail sufficient for accreditation. The clinical-grade tier, with its requirement for signed reproducibility bundles, BioCompute Objects, and external multi-site validation, is designed precisely to address this compliance challenge. Agent-native platforms must enforce tier boundaries, preventing agents from applying research-grade skills in clinical contexts without explicit override. A key implementation requirement is that agents must expose a deterministic replay mode: given a logged sequence of decisions, it must be possible to reproduce the exact execution path, even if the original selection was mediated by an LLM. This replay capability is essential for regulatory audit, debugging, and multi-site concordance testing.

To show that the framework is diagnostic rather than purely aspirational, Table 3 classifies the four surveyed systems against it, using only the validation evidence reported in their own publications and documentation. The exercise is deliberately conservative: a system is placed at the highest tier for which published evidence exists, not at the highest tier it could in principle reach. The result is that all four systems currently sit at the research-grade tier, with partial movement toward benchmarked validation, and none has published the external multi-site evidence that the clinical-grade tier requires. This is itself a finding: the ecosystem is generating analyses far faster than it is generating the validation evidence needed to trust them in consequential

Table 2. Proposed validation framework for agent-native genomic skills

Tier	Scope	Required validation	Acceptable risk	Governance
Research grade	hypothesis exploration, exploratory analysis	unit tests; synthetic adversarial inputs (empty files, malformed data, edge cases); minimal logging; researcher reviews all outputs	false positives tolerable if caught during interpretation	skill author responsible; community review encouraged
Benchmarked	publishable analyses, method comparison	all research-grade requirements plus validated performance on public reference datasets (e.g., GIAB for variant calling, curated scRNA-seq benchmarks for cell annotation); published metrics with confidence intervals; documented failure cases and known limitations	results must be reproducible across environments; known failure modes must be documented	independent benchmarking required; results published with skill metadata
Clinical grade	patient care, diagnostic reporting, pharmacogenomics	all benchmarked requirements plus external validation across independent sites; alignment with FDA/EMA regulatory guidelines for software as a medical device[29]; signed reproducibility bundles; audit trails conforming to BioCompute Objects[17]; version-locked dependencies; multi-site concordance testing	near-zero tolerance for false negatives in diagnostic contexts; all outputs must be traceable to specific skill versions and parameter sets	regulatory review required; CLIA/CAP compliance for clinical laboratories; agent behavior must be fully auditable

settings. These placements are provisional and rest solely on publicly available evidence; a system's tier should rise as its developers publish further validation.

Avoiding circular benchmark tuning

The benchmarked tier depends on the integrity of the reference datasets it draws on. Machine learning has repeatedly encountered a degenerate dynamic in which benchmarks intended as independent evaluations are progressively absorbed into the training distribution of the systems they are meant to evaluate, eroding their diagnostic value.[30] Agentic genomics will face the same pressure. As skill authors and agent developers compete to improve performance on public benchmarks (Genome in a Bottle for variant calling, curated scRNA-seq atlases for cell annotation, and CPIC guidelines for pharmacogenomic interpretation), the temptation to tune skills against the very benchmarks intended to evaluate them will be substantial. We recommend two structural protections. First, reference-dataset registries should retain held-out partitions that are not released until a system is submitted for evaluation, in the spirit of registered reports.[31] Second, benchmark contents should be rotated periodically, with the rotated partition retained for longitudinal calibration of pre-existing systems. Without these protections, the benchmarked tier risks degenerating into a leaderboard exercise that no longer measures generalization. The same logic applies to clinical-grade external validation: it is informative only to the extent that the external sites have not previously been used to tune the system under evaluation.

The framework aligns with existing standards. GA4GH data-sharing frameworks provide the infrastructure for interoperable skill metadata. FAIR principles ensure that skills are findable (through semantic registries), accessible (through open repositories), interoperable (through standardized input/output specifications), and reusable (through versioned, tested, documented modules). The nf-core community[1] has demonstrated that curated, community-reviewed bioinformatics pipelines can achieve high standards of reproducibility; agent-native skill registries should adopt analogous governance structures.

EQUITY AS A SYSTEMS DESIGN REQUIREMENT

The equity implications of agentic genomics are structural, not incidental. Because AI agents are optimized to find the most readily available and statistically abundant tools, reference datasets, and trained models, they will default to European-ancestry resources unless explicitly constrained. An agent asked to "run a polygenic risk score" will, in the absence of population-specific instructions, select models derived from predominantly European-ancestry genome-wide association studies (GWASs), producing scores that are miscalibrated for non-European populations. Agentic genomics therefore threatens to automate and accelerate existing biases[32] at an unprecedented scale.

Table 3. The four surveyed systems classified against the validation framework

System	Highest tier with published evidence	Validation evidence reported	Gap to next tier
CellAtria[13]	research grade, approaching benchmarked	standardized, reproducibility-first scRNA-seq pipeline; document-to-analysis reproducibility demonstrated	no published benchmarking with confidence intervals against public reference datasets
AutoBA[14]	research grade	automated code repair demonstrated; evaluations also revealed omitted steps and inappropriate tool choices	performance not yet quantified on public reference datasets with documented failure cases
Bio-Copilot[19]	research grade, approaching benchmarked	higher completion rates and fewer correction rounds than general-purpose LLMs on standardized omics workflows	benchmarks are internal; no independent multi-site reproduction published
ClawBio[15]	research grade	community adversarial testing; documented silent-failure incident and governance-layer remediation (this perspective); skills carry research/educational disclaimers	no external clinical validation; pharmacogenomic skills explicitly not offered as a medical device

This is not merely a problem of awareness. It is a systems design problem that requires engineering solutions at multiple levels.

Equity-aware default configurations

Agent-native skills that operate on population data must either include default parameters that are appropriate for diverse populations or require the user to specify population context before execution. A pharmacogenomic skill, for example, should default to querying population-specific allele frequency databases (e.g., gnomAD population stratifications and AfriCArGene for African pharmacogenomic variants) rather than defaulting to aggregate frequencies dominated by European-ancestry samples.

Population-aware model selection

When an agent selects a predictive model (for polygenic risk scoring, ancestry estimation, or phenotype prediction), the selection logic must account for the population composition of the training data. Skills should include metadata specifying which populations the underlying models were validated against, and agents should flag or refuse to apply models outside their validated population scope.

Bias detection during execution

Agents should monitor for signals of population mismatch during analysis: unexpected allele frequency distributions, anomalous principal-component projections, or calibration failures in predictive models, the last of which is well documented for polygenic risk scores applied across ancestries.[33] These checks can be implemented as automated quality-control steps within skills, analogous to the sequencing quality metrics that are standard in existing pipelines, and are a specific case of the out-of-distribution detection discussed above.

Measurable equity metrics

We propose that agent-native skill registries track and report (1) the population composition of validation datasets, (2) performance metrics stratified by ancestry group, and (3) the proportion of skills validated on non-European populations. These metrics make equity auditable rather than aspirational. This approach aligns with emerging frameworks for quantifying representational equity in biomedical data systems[32] and with efforts to develop health equity impact metrics that measure how computational tools perform across the populations they are applied to, not merely the populations they were trained on. We argue that equity metrics should be treated as first-class validation criteria, alongside accuracy and reproducibility: a skill that performs well on European-ancestry data but has not been evaluated on African or Latin American populations should not be considered fully validated, regardless of its technical performance on the populations it has been tested against.

The democratization potential of agentic genomics is particularly significant for researchers in low- and middle-income countries, where access to dedicated bioinformatics support is scarce.[32] Agent-native skill libraries, combined with local-first computation (where genetic data never leave the researcher's machine), could enable genomic analysis in settings that currently lack the infrastructure for it. Realizing this potential requires model hosting strategies that work with intermittent connectivity, documented minimum hardware specifications, and pre-packaged equity-aware defaults. Without these provisions, agentic genomics risks widening the very disparities it has the potential to close.

OPPORTUNITIES

Several structural benefits emerge when agents become the primary interface between researchers and bioinformatics tools.

Reproducibility by design

Agent-native skills encode their specifications explicitly: tools, versions, parameters, and reference data. When an agent executes a skill, it follows the same procedure every time. This addresses one of the most persistent problems in computational biology: more than 70% of researchers have reported being unable to reproduce another scientist's experiments.[34] Skills can include reproducibility bundles that capture every parameter and dependency, making replication straightforward. This property directly supports the reproducibility infrastructure that the broader computational biology community has been building through efforts such as nf-core, CWL, and BioCompute Objects.

Democratization and distributed expertise capture

Analyses that previously required specialized computational training, including pharmacogenomic interpretation, structural variant calling, and ancestry estimation, become accessible to any researcher who can describe their question in natural language. Simultaneously, open skill libraries enable domain experts to encode hard-won analytical knowledge into agent-executable modules without needing software engineering skills. A biologist who has spent years optimizing a variant-calling pipeline for a specific population can contribute that expertise directly. This is particularly significant for underrepresented populations, where the relevant domain knowledge often resides in precisely the research groups that have the least access to dedicated bioinformatics support. The result is a bidirectional democratization: broader access to advanced methods and broader participation in method development.

Accelerated hypothesis exploration

The ability to run multiple analyses in parallel, with agents operating autonomously on different questions, enables a more exploratory mode of science. Researchers can test speculative hypotheses that they would not have pursued under the time constraints of manual analysis. The same parallelism creates a multiple-comparisons hazard, which we address as agent-mediated p-hacking in the challenges section.

Hypothesis generation becomes the new bottleneck

When the cost of investigating a hypothesis collapses, the constraint on scientific progress shifts upstream to the hypothesis itself. A researcher who could previously test one well-motivated idea per month can now test ten in a week. The bottleneck becomes the quality and originality of the questions asked, not the capacity to answer them. This shift reframes the genomicist's role: comparative advantage shifts from analytical execution to careful biological reasoning, identification of underexplored phenomena, and the framing of testable claims. Agentic genomics will reward researchers who invest in the upstream activities of literature synthesis, model building, and counterintuitive question framing; downstream execution that has occupied much of the field's energy becomes a commodity.

Publishable workflows and agent-assisted peer review

The structured nature of agentic skills creates new possibilities for the publication and review of computational science. Workflows can be packaged as versioned, citable artifacts alongside the manuscripts they support: a paper reporting a polygenic risk score can deposit the exact skill invocation sequence, parameters, and reference data used to generate the result, with a digital object identifier that survives independent of the underlying agent platform.[17] Reviewers can then re-execute the analysis on the deposited workflow, perturb its parameters, or apply it to fresh data, in a way that text-based methods sections do not support. Agents themselves can act as reviewer aids: an agent given a manuscript together with its associated workflow can flag inconsistencies between the prose and the executed analysis, recompute headline statistics, and test the robustness of conclusions to alternative parameter choices. We do not view this as a replacement for human peer review but as a structural augmentation that closes a gap the field has tolerated for decades.

Preregistration becomes operationally lightweight

The barriers to preregistration in computational biology have historically been practical rather than philosophical: writing a complete analytical plan in prose, deviating from it during analysis, and documenting deviations transparently is laborious. Agent-native workflows reshape this calculus. A preregistered analysis can be expressed as a specific sequence of skill invocations with fixed parameters, deposited at a registry such as the Open Science Framework prior to data acquisition, and executed verbatim once data are available.[31] Deviations from the plan become automatically detectable as differences between the registered and executed skill traces. This operationalizes preregistration in a way that prose protocols cannot and provides a structural answer to the p-hacking risk identified in the challenges section.

CHALLENGES

The challenges are equally structural, and some are not yet fully understood.

Validation at scale

The rate of output generation now exceeds the rate of human validation. The tiered framework proposed above provides a structure, but its implementation requires community consensus on benchmarks, governance of skill registries, and investment in adversarial testing infrastructure.

Agent-mediated p-hacking and exploration inflation

The same property that makes agentic genomics attractive, the ability to run many analytical variants in parallel without proportional human effort, creates a new vector for inadvertent or deliberate p-hacking.[35] A researcher exploring a dataset can now instruct an agent to run a dozen variants, each subtly differing in covariate adjustment, filtering threshold, or population subset, and report the most striking result. The opportunity cost of running each variant collapses; the multiple-comparison burden does not. Without preregistration of analytical plans and explicit accounting for the number of hypotheses tested, agentic workflows will amplify existing problems with false discovery at a rate faster than the field has demonstrated it can respond to.

Silent-failure modes

The most dangerous errors in agentic genomics are those that produce plausible-looking output from flawed analyses. An agent might silently mix GRCh37 and GRCh38 genomic coordinates when chaining tools that default to different reference assemblies, producing variant calls at incorrect positions that pass format validation but yield biologically meaningless results. An agent configuring an alignment pipeline might silently drop reads that fail quality thresholds without flagging the resulting loss of coverage. Detecting these failures requires domain expertise, adversarial test suites, and a culture of transparent reporting.

Clinical safety and regulatory fit

The application of agentic genomics in clinical settings raises unresolved regulatory questions. AI agents that autonomously select and configure pipelines introduce non-deterministic behavior that sits uneasily within existing regulatory categories for software as a medical device.[29,36] Regulatory bodies, including the FDA and EMA, have frameworks for deterministic clinical software with fixed, auditable logic. An agent whose behavior is mediated by an LLM requires new regulatory approaches that preserve auditability while accommodating the flexibility that makes agents useful.

Intellectual provenance

When an agent generates a pipeline by combining elements from multiple sources, the intellectual provenance of the resulting analysis becomes unclear. Attribution, reproducibility, and credit assignment all require new frameworks in an agent-mediated research environment. Skill registries with DOI-minted releases, version-controlled specifications, and formal metadata will be essential for establishing provenance chains.

The expertise paradox

Agentic genomics expands access to computational methods but does not transfer the judgment needed to evaluate their outputs. Training the next generation of genomicists as orchestrators and validators rather than coders is a pedagogical challenge that the field has not yet addressed. Graduate programs and bioinformatics curricula will need to emphasize critical evaluation of automated outputs alongside (or instead of) manual pipeline construction.

Entanglement with the broader scientific culture

Agentic genomics enters a research ecosystem that already struggles with open data sharing, reproducibility, p-hacking, peer-review capacity, and credit assignment. It will inherit these problems and amplify some of them. None of the structural mitigations proposed in this perspective, including the tiered validation framework, preregistration of agent workflows, and standardized skill specifications, can succeed in isolation from broader scientific-culture reforms. The agentic paradigm is therefore best understood not as a self-contained technical shift but as one element in a longer-running effort to make computational biology rigorous, transparent, and equitable; its success will be measured in part by whether it accelerates or impedes that broader program.

A FRAMEWORK FOR RESPONSIBLE ADOPTION

We propose five principles for the responsible adoption of agentic genomics.

First, domain expertise remains the irreducible requirement. AI agents amplify the researcher's capacity; they do not replace the researcher's judgment. Every result produced by an agent must be evaluated by someone with sufficient domain knowledge to detect errors. This principle should inform institutional decisions about staffing, training, and resource allocation.

Second, validation must be proportional to consequence. The three-tier framework (research grade, benchmarked, and clinical grade) provides a structure for calibrating scrutiny to risk. Agent-native platforms should enforce tier boundaries and make validation status visible to users at the point of execution.

Third, transparency is non-negotiable. When an AI-generated analysis fails, the failure must be reported openly and fixed publicly. Defensive responses to bug reports undermine the trust that community-driven development depends on. This applies equally to academic and commercial developers of agentic systems.

Fourth, skills must be testable by design. Agent-native skills should include test suites that cover normal operation, edge cases, and adversarial inputs. Automated testing should run on every update. Skills that lack tests should be flagged as unvalidated. This is no different in principle from the testing expectations for any software that generates scientific results; the difference is that the speed of agentic development makes untested code easier to produce and harder to catch.

Fifth, equity must be engineered, not declared. Population-aware defaults, bias detection mechanisms, and equity metrics in skill registries are engineering requirements, not aspirational statements. The field should treat the absence of equity-aware design with the same seriousness it treats the absence of input validation.

CONCLUSION

Agentic genomics is not a future possibility. It is a present reality, adopted unevenly but advancing rapidly. The combination of LLMs, autonomous agents, and domain-specific skill libraries is changing who can do genomics, how fast they can do it, and what kinds of questions they can ask.

The central thesis of this perspective is that this shift moves the primary bottleneck from pipeline construction to validation. The consequences of that shift are far-reaching: validation frameworks must be formalized and tiered; equity must be engineered into default configurations; regulatory frameworks must accommodate non-deterministic, LLM-mediated software; and the role of domain expertise must be redefined as the irreducible human contribution to an increasingly automated science.

The opportunity is substantial: more reproducible analyses, broader access to advanced methods, faster iteration, and a new model of community-driven scientific software development. The risks are equally real: silent failures, hallucinated results, clinical safety concerns, automated amplification of population biases, and a growing divide between researchers who have adopted these methods and those who have not.

The path forward is to build the institutional, technical, and cultural infrastructure that ensures AI-generated genomic analyses are trustworthy. That infrastructure starts with domain expertise, the one thing that cannot be automated, and extends through validation frameworks, equity-aware engineering, transparent communities, and a clear-eyed understanding of what these tools can and cannot do. As with the transition from manual to automated sequencing, the question is no longer whether agentic genomics will be adopted but whether the field will establish the standards required to make it trustworthy before it becomes ubiquitous.

METHODS

This perspective analyses previously published systems and does not report new sequencing data. The only procedure executed for this work is the perturbation test of the ClawBio pharmacogenomic skill reported in the section the validation bottleneck: the new rate limiting step. Its protocol is summarized below; full input descriptions, a key resources table, and notes on the qualitative syntheses in Box 1 and Tables 1 and 3 are provided in the supplemental information.

Perturbation test

The perturbation result reported in the section the validation bottleneck: the new rate limiting step was executed on June 2, 2026, using ClawBio's pharmgx-reporter skill (v.0.1.0). The skill was run on three perturbed inputs (an empty file, a file containing non-genotype content, and a comment-only file with no variant rows) and, as a control, one valid 23andMe-format genotype file. Each perturbed input caused the skill to halt with an explicit file-format detection error and a non-zero exit status, whereas the valid input produced a complete report. This reproduces, on the current release, the perturbation that an earlier community audit found to produce a silent all-normal pharmacogenomic report. Full input descriptions are given in the supplemental information.

ACKNOWLEDGMENTS

We thank the contributor communities of the open agentic genomics projects discussed here for stress-testing these systems in public and, in particular, for demonstrating that transparent, community-driven development can surface and correct failures that closed development would hide. Their audits have informed the discussion of validation in this perspective.

DECLARATION OF INTERESTS

M.C. is the creator and maintainer of ClawBio. At the time of writing, M.C. and H.G. were associated with GENEQ Global Ltd.

SUPPLEMENTAL INFORMATION

Supplemental information can be found online at https://doi.org/10.1016/j.xgen.2026.101305.

REFERENCES

1. Ewels, P.A., Peltzer, A., Fillinger, S., Patel, H., Alneberg, J., Wilm, A., Garcia, M.U., Di Tommaso, P., and Nahnsen, S. (2020). The nf-core framework for community-curated bioinformatics pipelines. Nat. Biotechnol. *38*, 276–278. https://doi.org/10.1038/s41587-020-0439-x.
2. Mölder, F., Jablonski, K.P., Letcher, B., Hall, M.B., Tomkins-Tinch, C.H., Sochat, V., Forster, J., Lee, S., Twardziok, S.O., Kanitz, A., et al. (2021). Sustainable data analysis with Snakemake. F1000Res. *10*, 33. https://doi.org/10.12688/f1000research.29032.2.
3. Abueg, L.A.L., Afgan, E., Allart, O., Awan, A.H., Bacon, W.A., Baker, D., Bassetti, M., Batut, B., Bernt, M., Blankenberg, D., et al. (2024). The Galaxy platform for accessible, reproducible, and collaborative data analyses: 2024 update. Nucleic Acids Res. *52*, W83–W94. https://doi.org/10.1093/nar/gkae410.
4. Karpathy, A. (2025). Vibe coding. https://x.com/karpathy/status/1886192184808149383 (accessed 4 April 2026).
5. Thirunavukarasu, A.J., Ting, D.S.J., Elangovan, K., Gutierrez, L., Tan, T.F., and Ting, D.S.W. (2023). Large language models in medicine. Nat. Med. *29*, 1930–1940. https://doi.org/10.1038/s41591-023-02448-8.
6. Tian, S., Jin, Q., Yeganova, L., Lai, P.-T., Zhu, Q., Chen, X., Yang, Y., Chen, Q., Kim, W., Comeau, D.C., et al. (2024). Opportunities and challenges for ChatGPT and large language models in biomedicine and health. Brief. Bioinform. *25*, bbad493. https://doi.org/10.1093/bib/bbad493.
7. Dagdelen, J., Dunn, A., Lee, S., Walker, N., Rosen, A.S., Ceder, G., Persson, K.A., and Jain, A. (2024). Structured information extraction from scientific text with large language models. Nat. Commun. *15*, 1418. https://doi.org/10.1038/s41467-024-45563-x.
8. Wang, L., Ma, C., Feng, X., Zhang, Z., Yang, H., Zhang, J., Chen, Z., Tang, J., Chen, X., Lin, Y., et al. (2024). A survey on large language model based autonomous agents. Front. Comput. Sci. *18*, 186345. https://doi.org/10.1007/s11704-024-40231-1.
9. Boiko, D.A., MacKnight, R., Kline, B., and Gomes, G. (2023). Autonomous chemical research with large language models. Nature *624*, 570–578. https://doi.org/10.1038/s41586-023-06792-0.
10. Dip, S.A., Mallick, D., Acharjee Shuvo, U., Barua Soumma, S., Rafsani, F., Kumar Paul, B., Ahmed Moumi, N., Ahmed, S., and Zhang, L. (2026). Large language model agents for biological intelligence across genomics, proteomics, spatial biology, and biomedicine. Brief. Bioinform. *27*, bbag110. https://doi.org/10.1093/bib/bbag110.
11. Qi, C., Wang, W., Jiang, S., Liu, Q., Song, X., Fang, H., and Wei, Z. (2026). Artificial intelligence agents for biological research: a survey. Brief. Bioinform. *27*, bbag075. https://doi.org/10.1093/bib/bbag075.
12. Zhou, J., Jiang, J., Han, Z., Wang, Z., and Gao, X. (2025). Streamline automated biomedical discoveries with agentic bioinformatics. Brief. Bioinform. *26*, bbaf505. https://doi.org/10.1093/bib/bbaf505.
13. Nouri, N., Artzi, R., and Savova, V. (2026). An agentic AI framework for ingestion and standardization of single-cell RNA-seq data analysis. npj Artif. Intell. *2*, 8. https://doi.org/10.1038/s44387-025-00064-0.
14. Zhou, J., Zhang, B., Li, G., Chen, X., Li, H., Xu, X., Chen, S., He, W., Xu, C., Liu, L., and Gao, X. (2024). An AI agent for fully automated multi-omic analyses. Adv. Sci. *11*, 2407094. https://doi.org/10.1002/advs.202407094.
15. Corpas, M. (2026). ClawBio: Bioinformatics-Native AI Agent Skill Library (v0.5.0). Zenodo. https://doi.org/10.5281/zenodo.19420648.
16. Peng, S., Kalliamvakou, E., Cihon, P., and Demirer, M. (2023). The impact of AI on developer productivity: evidence from GitHub Copilot. Preprint at arXiv. https://doi.org/10.48550/arXiv.2302.06590.
17. Alterovitz, G., Dean, D., Goble, C., Crusoe, M.R., Soiland-Reyes, S., Bell, A., Hayes, A., Suresh, A., Purkayastha, A., King, C.H., et al. (2018). Enabling precision medicine via standard communication of HTS provenance, analysis, and results. PLoS Biol. *16*, e3000099. https://doi.org/10.1371/journal.pbio.3000099.
18. Wilkinson, M.D., Dumontier, M., Aalbersberg, I.J., Appleton, G., Axton, M., Baak, A., Blomberg, N., Boiten, J.-W., da Silva Santos, L.B., Bourne, P.E., et al. (2016). The FAIR Guiding Principles for scientific data management and stewardship. Sci. Data *3*, 160018. https://doi.org/10.1038/sdata.2016.18.
19. Liu, Y., Shen, R., Zhou, L., Xiao, Q., Yuan, J., and Li, Y. (2025). A data-intelligence-intensive bioinformatics copilot system for large-scale omics research and scientific insights. Brief. Bioinform. *26*, bbaf312. https://doi.org/10.1093/bib/bbaf312.
20. Tang, X., Qian, B., Gao, R., Chen, J., Chen, X., and Gerstein, M.B. (2024). BioCoder: a benchmark for bioinformatics code generation with large language models. Bioinformatics *40*, i266–i276. https://doi.org/10.1093/bioinformatics/btae230.
21. Jin, Q., Yang, Y., Chen, Q., and Lu, Z. (2024). GeneGPT: augmenting large language models with domain tools for improved access to biomedical information. Bioinformatics *40*, btae075. https://doi.org/10.1093/bioinformatics/btae075.
22. Wang, Z., Jin, Q., Wei, C.-H., Tian, S., Lai, P.-T., Zhu, Q., Day, C.-P., Ross, C., Leaman, R., and Lu, Z. (2025). GeneAgent: self-verification language agent for gene-set analysis using domain databases. Nat. Methods *22*, 1677–1685. https://doi.org/10.1038/s41592-025-02748-6.
23. Dong, Z., Zhong, V., and Lu, Y. (2023). BioMANIA: simplifying bioinformatics data analysis through conversation. Preprint at bioRxiv. https://doi.org/10.1101/2023.10.29.564479.

24. Huang, K., Zhang, S., Wang, H., Qu, Y., Lu, Y., Roohani, Y., Li, R., Qiu, L., Li, G., Zhang, J., et al. (2025). Biomni: a general-purpose biomedical AI agent. Preprint at bioRxiv. https://doi.org/10.1101/2025.05.30.656746.

25. Goh, E., Gallo, R., Hom, J., Strong, E., Weng, Y., Kerman, H., Cool, J.A., Kanjee, Z., Parsons, A.S., Ahuja, N., et al. (2024). Large language model influence on diagnostic reasoning: a randomized clinical trial. JAMA Netw. Open *7*, e2440969. https://doi.org/10.1001/jamanetworkopen.2024.40969.

26. Hendrycks, D., and Gimpel, K. (2017). A baseline for detecting misclassified and out-of-distribution examples in neural networks. In Proceedings of the 5th International Conference on Learning Representations (ICLR). https://openreview.net/forum?id=Hkg4TI9xl.

27. Farquhar, S., Kossen, J., Kuhn, L., and Gal, Y. (2024). Detecting hallucinations in large language models using semantic entropy. Nature *630*, 625–630. https://doi.org/10.1038/s41586-024-07421-0.

28. Singhal, K., Azizi, S., Tu, T., Mahdavi, S.S., Wei, J., Chung, H.W., Scales, N., Tanwani, A., Cole-Lewis, H., Pfohl, S., et al. (2023). Large language models encode clinical knowledge. Nature *620*, 172–180. https://doi.org/10.1038/s41586-023-06291-2.

29. Gerke, S., Babic, B., Evgeniou, T., and Cohen, I.G. (2020). The need for a system view to regulate artificial intelligence/machine learning-based software as medical device. npj Digit. Med. *3*, 53. https://doi.org/10.1038/s41746-020-0262-2.

30. Recht, B., Roelofs, R., Schmidt, L., and Shankar, V. (2019). Do ImageNet classifiers generalize to ImageNet? In Proceedings of the 36th International Conference on Machine Learning, *97* (PMLR), pp. 5389–5400. https://proceedings.mlr.press/v97/recht19a.html.

31. Nosek, B.A., Ebersole, C.R., DeHaven, A.C., and Mellor, D.T. (2018). The preregistration revolution. Proc. Natl. Acad. Sci. USA *115*, 2600–2606. https://doi.org/10.1073/pnas.1708274114.

32. Corpas, M., Freidin, M.B., Valdivia-Silva, J., Baker, S., Fatumo, S., and Guio, H. (2026). Three dimensions of compounding neglect: how biobanks, clinical trials, and scientific literature systematically exclude the Global South. Preprint at medRxiv. https://doi.org/10.64898/2026.02.10.26346004.

33. Martin, A.R., Kanai, M., Kamatani, Y., Okada, Y., Neale, B.M., and Daly, M.J. (2019). Clinical use of current polygenic risk scores may exacerbate health disparities. Nat. Genet. *51*, 584–591. https://doi.org/10.1038/s41588-019-0379-x.

34. Baker, M. (2016). 1,500 scientists lift the lid on reproducibility. Nature *533*, 452–454. https://doi.org/10.1038/533452a.

35. Head, M.L., Holman, L., Lanfear, R., Kahn, A.T., and Jennions, M.D. (2015). The extent and consequences of p-hacking in science. PLoS Biol. *13*, e1002106. https://doi.org/10.1371/journal.pbio.1002106.

36. Topol, E.J. (2019). High-performance medicine: the convergence of human and artificial intelligence. Nat. Med. *25*, 44–56. https://doi.org/10.1038/s41591-018-0300-7.

Cell Genomics, Volume *6*

Supplemental information

Agentic genomics: From pipeline automation to autonomous validation

Manuel Corpas, Heinner Guio, and Segun Fatumo

Supplemental Information

Agentic Genomics: From Pipeline Automation to Autonomous Validation

Manuel Corpas, Segun Fatumo, Heinner Guio

This document provides supplemental material referenced in the main text: a key resources table (Table S1), the full description of the perturbation test inputs (Note S1), and notes on the basis of the qualitative syntheses presented in Box 1 and Tables 1 and 3 (Notes S2 and S3).

Table S1. Key resources.

Resource	Source	Identifier
ClawBio skill library	Corpas (2026)	https://doi.org/10.5281/zenodo.19420648
pharmgx-reporter skill (perturbation test)	ClawBio	version 0.1.0

Note S1. Perturbation test inputs.

The perturbation result reported in the validation section of the main text was executed on 2 June 2026 using ClawBio's pharmgx-reporter skill (version 0.1.0). The skill was run on three perturbed inputs and, as a control, one valid input:

1. An empty file (zero bytes, no genomic data).
2. A file containing non-genotype content (text that does not conform to any recognised genotype format).
3. A comment-only file with header or comment lines but no variant rows.
4. As a control, one valid 23andMe-format genotype file.

Each perturbed input caused the skill to halt with an explicit file-format detection error and a non-zero exit status, whereas the valid input produced a complete report. This reproduces, on the current release, the perturbation that an earlier community audit found to produce a silent "all normal" pharmacogenomic report. The silent-failure path exposed by the original audit has been closed by the governance-layer remediation process described in the main text: community audit, public disclosure, and a fix that converts a silent degradation into a loud, diagnosable failure.

Note S2. The Box 1 illustrative example.

Box 1 in the main text is an illustrative example, not a benchmark or a logged experiment. It describes, qualitatively, the pattern we have observed when an AI agent orchestrates standard open-source tools (fastp for adapter trimming and quality control, BWA-MEM2 for alignment to GRCh38, GATK HaplotypeCaller and DeepVariant for variant calling, and SnpEff with ClinVar for annotation) on a local workstation for trio exome analysis. We did not conduct a systematic benchmark for this Perspective; Box 1 reports no timings, counts, or comparative measurements, and no claim of measured performance is made. Controlled, logged, multi-site benchmarking with confidence intervals is identified in the main text as a priority for the field rather than a result of this work.

Note S3. Tables 1 and 3.

The values in Table 1 are a qualitative synthesis drawn from the cited reports and contain no new measurements. The tier classification in Table 3 was derived solely from the validation evidence described in each system's cited publication, with each system placed at the highest tier for which published evidence exists.
